# *Fasciola hepatica*: the dispersal of cercariae shed by the snail *Galba truncatula*

**DOI:** 10.1051/parasite/2020013

**Published:** 2020-03-18

**Authors:** Daniel Rondelaud, Philippe Vignoles, Gilles Dreyfuss

**Affiliations:** 1 Laboratory of Parasitology, Faculty of Pharmacy 2 Rue du Docteur Raymond Marcland 87025 Limoges Cedex France

**Keywords:** *Fasciola hepatica*, Limousin, Metacercaria, *Nasturtium officinale*, Watercress bed

## Abstract

Field investigations in 14 wild watercress beds located in the French region of Limousin, a known endemic area for distomatosis, were performed for three years to determine the distance that cercariae of *Fasciola hepatica* can reach in water before their encystment on the host plant. Each bed was located on the course of an open drainage furrow, while snails (*Galba truncatula*) lived upstream around the emergence of a source. Five plant species were collected in early April and examined to find metacercariae. Most cysts were noted on *Nasturtium officinale* (188 on 48.7 kg of dripped plants), followed by *Helosciadium nodiflorum* (125 on 33.4 kg). On the other plant species, there were few larvae. Most cercariae encysted on the plants growing in the most upstream part of each bed, usually on the first 50 cm in length. When water in the beds was fast running, the distribution of metacercariae was more limited and their number was fewer than those in the beds fed by a slow flow of water. Cercariae were able to swim or were carried away by the current up to a mean of 5 m in slow-flow waters before encysting; this distance was only 4 m in faster waters. Plants growing on the most upstream section of a watercress bed located in a drainage furrow are the most used by cercariae for their encystment, when snails live around the emergence of a source. The speed of the water current affected the number and distribution of metacercariae in the bed.

## Abstract (fr)

Des investigations de terrain dans 14 cressonnières naturelles localisées dans la région française du Limousin, zone connue d’endémie de distomatose, ont été effectuées pendant trois années pour déterminer la distance que les cercaires de *Fasciola hepatica* peuvent atteindre dans l’eau avant leur enkystement sur la plante hôte. Chaque cressonnière était située sur le cours d’une rigole de drainage superficiel, tandis que les mollusques (*Galba truncatula*) vivaient plus en amont autour de l’émergence d’une source. Cinq espèces végétales ont été recueillies au début d’avril et examinées pour y trouver des métacercaires. La plupart des kystes ont été observés sur *Nasturtium officinale* (188 sur 48,7 kg de plantes égouttées), suivis par *Helosciadium nodiflorum* (125 sur 33,4 kg). Sur les autres espèces végétales, il y avait peu de larves. La plupart des cercaires étaient enkystées sur les plantes qui poussaient dans la partie la plus en amont de chaque cressonnière (habituellement sur les premiers 50 cm de longueur). Lorsque l’eau courante dans les cressonnières était rapide, la distribution des métacercaires était plus limitée et leur nombre était inférieur à celui observé dans les stations alimentées par un débit d’eau lent. Les cercaires ont été capables de nager ou ont été entrainées par le courant jusqu’à 5 m en moyenne dans une eau à écoulement lent avant leur enkystement ; cette distance n’était que de 4 m dans les eaux plus rapides. Les plantes qui poussent sur la section la plus en amont d’une cressonnière située dans une rigole de drainage sont les plus utilisées par les cercaires pour leur enkystement lorsque les mollusques vivent autour de l’émergence d’une source. La vitesse du courant d’eau affecte le nombre et la distribution des métacercaires dans la cressonnière.

## Introduction

In Western Europe, human distomatosis caused by the digenean *Fasciola hepatica* Linnaeus, 1758 [10] is often due to consumption of watercress (*Nasturtium officinale* W.T. Aiton, 1812 [1]) [11–13]. Wild watercress was reported to be the most common infected plant in surveys performed by Rondelaud [19] and Rondelaud et al. [21] in patients affected by fasciolosis in the French region of Limousin, a known endemic area. According to Rondelaud et al. [21], consumption of this plant was reported by 516 patients (out of a total of 616 infected people) between 1955 and 1998. Cercariae of *F. hepatica* often use wild watercress for their encystment. Their location on the plant is dependent not only on the ability of cercariae to locate the host plant, but also on the distribution and mobility of the host snail [14]. The majority of cercariae encysted just below the water surface, typically less than 2 cm depth [4, 24]. According to Pécheur [16], the metacercariae are encysted to the upper or lower surface of the leaves depending on the plant species, while Hodasi [7] observed them mainly on the underside of submerged leaves. The green parts of plants rather than the brown and decaying parts are preferred by these metacercarial cysts [7].

On the acidic soils of Limousin, a total of 252 wild watercress beds as sources of human distomatosis cases were investigated by our team between 1970 and 2000 [18, 19, 21, 22]. Generally small in area (<2 m^2^), most of these beds are located outside the pastures grazed by cattle or sheep, and 52% of them are isolated by a fence or a wall, thus preventing any contact with domestic or wild mammals. In these watercress beds, *F. hepatica* infections were low with a mean metacercarial burden of 2.6–6.3 per bed and a wide annual variation in the number of infected beds [5]. The distribution of *F. hepatica* metacercariae did not appear to favour a plant species, with an almost equal number of parasites being recovered on both *N. officinale* and false watercress (*Helosciadium nodiflorum* (Linnaeus) W.D.J. Koch, 1824 [8]) [5]. Despite the results reported in the quantitative studies of Dreyfuss et al. [5] in the field, there is still a gap in our knowledge of the relationships between metacercariae and their host plants. For example, little information is available in the literature on the distance that the cercariae of *F. hepatica* can travel from the host snail before encysting on the host plant [2]. In view of this situation, the following three questions arose: how were the cercariae of *F. hepatica* dispersed and encysted in the watercress bed when the population of the host snail lived away from it? What was the distance that cercariae can reach after their release from the host snail? Could this distance vary according to the speed of the current flowing in the bed? To answer these three questions, field investigations were carried out in a total of 14 watercress beds located on the acidic soils of Limousin. As contamination of beds by the metacercariae of *F. hepatica* was irregular over time [4], the investigations were carried out for three years (2016–2018) in order to have a significant number of metacercariae counted in each type of bed.

## Materials and methods

### Watercress beds studied

**Figure 1:**
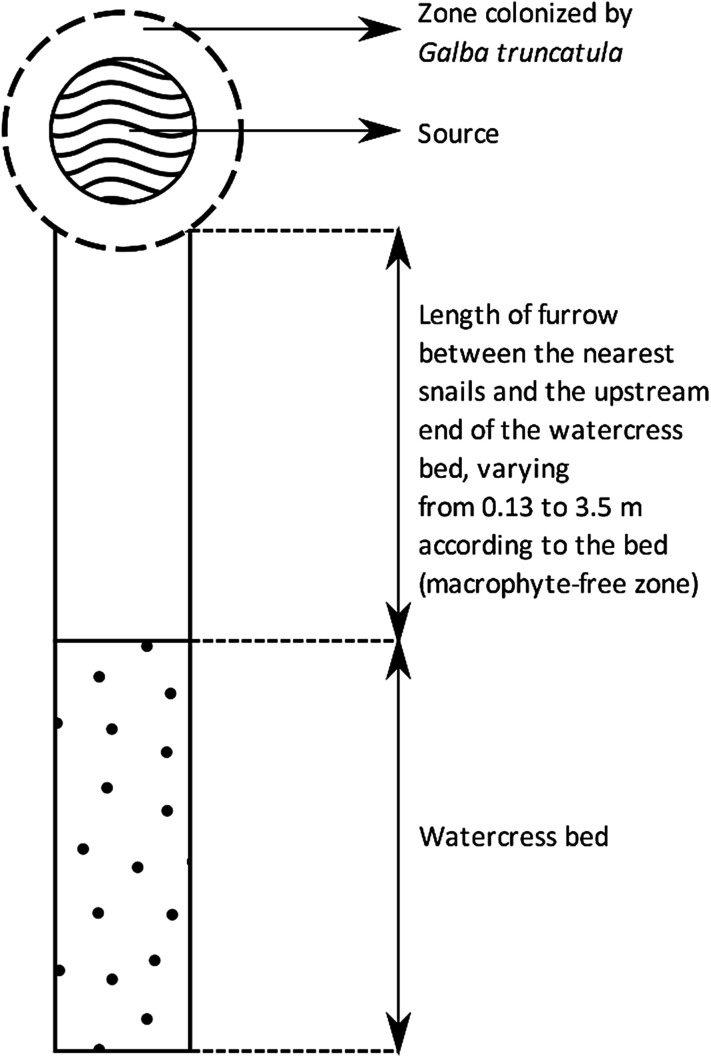
Schematic representation of the upstream end of a surface drainage furrow studied. This drawing shows the area occupied by the snails around the source, the area without macrophytes, of variable extent depending on the furrow, and the watercress bed located further downstream. The dimensions of these various zones have not been indicated on this drawing because they vary according to the type of bed and the length of the zone without macrophytes.

A total of 14 watercress beds located in the French administrative department of Haute Vienne were selected for this study. They were situated in surface drainage furrows present in 14 grasslands on granite or gneiss. Table 1 indicates the number of beds studied according to their type, length and area. The speed of water current during our investigations in early April is also indicated. They were selected according to the following two criteria: (i) many metacercariae were counted on watercress in these wild beds between 1990 and 2004 [5, 20]; (ii) the population of the host snail: *Galba truncatula* (O.F. Müller, 1774 [15]), was living around the emergence of a source that flowed in an open drainage furrow, while the bed was downstream on the course of the furrow, at a distance of 0.1–0.3 m (5 beds), 1.1–1.8 m (4 beds) or 3.0–3.5 m (5 beds) from the nearest snails. This distance is called “furrow length” in the body of this paper (Fig. 1).

**Table 1:** Main characteristics of the 14 watercress beds studied in 2016–2018 in northern Haute Vienne, with indication of the number of *Fasciola hepatica* metacercariae.

Type of watercress bed	Length of the macrophyte-free zone (m)	Number of beds	Length of the bed in m (area in m^2^)	Flow speed (cm/s)*	Number of cysts over 3 years	
					Total	Means (SD)
With a slow-flow spring	0.13–0.24	3	6.6–9.2 (2.9–4.1)	0.5–1.7	89	29.6 (17.3)
	1.1–1.8	2	6.3–7.9 (2.8–3.5)	0.9–2.1	81	40.5 (27.5)
	3.1–3.5	2	7.8–9.5 (3.5–4.2)	0.7–1.6	50	25.0 (12.5)
With a faster-flow spring	0.15–0.29	2	6.4–6.9 (2.8–3.1)	5.2–6.7	93	46.5 (15.2)
	1.3–1.6	2	8.1–11.2 (3.6–5.0)	4.3–7.2	51	25.5 (8.4)
	3.0–3.2	3	9.3–12.2 (4.1–5.4)	5.4–8.2	4	1.3 (0.4)

* Two records per year and per furrow over a three-year period. The records were collected in early April with a 3-day interval between 10 a.m. and 11 a.m.

The first seven beds were each fed by a slow-flow spring. Their width did not exceed 45 cm for a mean depth of 20 cm, and their sediment was largely muddy sometimes with gravel and sand. The seven other beds were each fed by a faster-flowing spring. They had the same size as above, but their depth was greater (with a mean of 30 cm). Their sediment was mainly sandy with rare muddy beaches. In both bed types, the area of snail habitat ranged from 1.2 m^2^ to 1.7 m^2^ in April 2016, while the number of overwintering snails ranged from 18 to 31 adults. No difference between these areas or between these numbers was noted, regardless of the type of watercress bed (with a slow-flow or a faster-flow spring). The pH of running water ranged from 6.2 to 7, while the concentration of calcium ions was less than 20 mg/L [3, 6]. In each of the 14 beds, the watercress tufts were few in number and were distributed along the course of each furrow. Other macrophytes such as *H. nodiflorum*: 12 beds, *Juncus effusus* Linnaeus, 1753 [9]: 11 beds, *Veronica beccabunga* Linnaeus, 1753 [9]: 7 beds, and *Callitriche* sp.: 5 beds, were also present but in low numbers.

The continental type climate is strongly modulated by humid winds coming from the Atlantic Ocean. Depending on the year, the average annual rainfall varied from 800 mm to 1000 mm, while the average annual temperature ranged from 3.1 °C to 4.1 °C in January and from 18.6 °C to 19.3 °C in July, depending on the municipalities (https://fr.climate-data.org).

### Protocol of investigations

In each bed, the rare macrophytes present in the furrow between the snail population and the first tuft of watercress were torn off each year in early February in order to have a macrophyte-free zone between snails and the bed downstream (Fig. 1). Investigations were then carried out in early April after the cercarial shedding of overwintering snails (usually in mid-March). At each investigation date, research focused on five plant species: *Callitriche* sp., *H. nodiflorum*, *J. effusus*, *N. officinale*, and *V. beccabunga*. These species were selected because of their smooth surfaces which allow good adhesion of larvae to the plant [7], and their presence in several watercress beds as indicated above.

The distance between the centre of each plant (with a single or several stems) and the upstream end of each bed was first measured. The position of each plant in relation to the width of the drainage furrow was calculated subsequently. The submerged and emerged parts were then cut at the plant neck and transported to the laboratory in an individual bag with a label indicating the plant species and distance values. In the laboratory, stems and leaves were placed for 24 h at room temperature for them to drip before being weighed. They were then examined under a stereomicroscope to search for metacercariae of *F. hepatica* or other digeneans. The morphological criteria for differentiating these metacercariae from those of other Digenea have already been specified in earlier papers [4, 5].

### Parameters studied

Individual values noted for metacercariae in each type of bed over the three years of investigations were pooled in order to obtain fairly high values because the number of metacercariae is generally low in the wild beds of Limousin [5, 20].

The first parameter was the number of *F. hepatica* cysts found in each type of watercress bed and took into account the length of the macrophyte-free zone. Two other parameters were the total number of cysts on each host plant per year and that of larvae per kg of host plant. Individual values noted for the first or the third parameters were averaged and standard deviations calculated. These data were analysed using the Shapiro–Wilk normality test [23]. As the distributions of these values were not normal, the Kruskal–Wallis test was used to establish levels of significance.

The relative distance between each plant carrying metacercariae and the upstream end of each bed was the fourth parameter, and was expressed as 50-cm long sections in order to classify these larvae relative to the position of each plant in the bed. The last parameter was the position of each plant in relation to the width of the bed (45 cm), and was expressed as 15-cm long sections. Individual values noted for either parameter were averaged and standard deviations were calculated, taking into account the type of watercress bed and the length of the macrophyte-free zone. These values were also analysed using the Shapiro–Wilk normality test [23]. As the distributions of these values were not normal, the Scheirer–Ray–Hare test, completed by Siegel and Castellan’s *post hoc* test, was used to establish levels of significance.

All the analyses were performed using R 3.3.0 software [17].

## Results

A total of 368 metacercariae, i.e. 220 in the furrows with a slow-flow spring and 148 in those with a faster-flow spring, were noted in the 14 watercress beds during the three years of study.

### Distribution of metacercariae on grass

Table 1 shows the number of metacercariae of *F. hepatica* found in each type of bed, taking into account the length of the macrophyte-free zone. In beds fed by a slow-flow spring, the mean number of cysts per bed ranged from 25.0 to 40.5, and no difference between these mean numbers was noted. In beds fed by a faster-flow spring, there was a decrease in the mean numbers of cysts (from 46.5 to 1.3 larvae, respectively) when the length of the macrophyte-free zone between the nearest snails and the bed increased, but this variation was not significant.

The distribution of these metacercariae for each host plant species is given in Table 2. Most metacercariae were found on *N. officinale*: a total of 115 in beds fed by slow-flow running water, and 73 in the others fed by a faster flow of water. The number of these larvae was lower on *H. nodiflorum*: 63 and 62 cysts, respectively. On the other three plant species, this number was low: from 13 to 16 cysts in the beds with a low flow of water, from 1 to 6 in the other beds. The average metacercarial load per kg of drained plants was close in *Callitriche* sp., *H. nodiflorum*, and *N. officinale* (36–3.8 cysts/kg), while it was significantly lower (*H*_4_ = 35.52, *p* < 0.001) in *J. effusus* (1.9 kg^−1^) and *V. beccabunga* (1.0 kg^−1^).

**Table 2:** Number of *Fasciola hepatica* metacercariae and weight of dripped plants in relation to the type of watercress bed and the year. The number of cysts per kg of plants was calculated on all metacercariae, without taking into account the type of bed.

Parameter, type of bed and year	*Nasturtium officinale*	*Callitriche* sp.	*Helosciadium nodiflorum*	*Juncus effusus*	*Veronica beccabunga*
Total number of cysts (plants in kg)					
Slow-flow spring					
2016	49 (10.3)	9.1 (1.2)	29 (7.2)	3 (2.4)	5 (2.2)
2017	27 (8.7)	3 (1.1)	19 (5.5)	8 (1.2)	7 (2.6)
2018	39 (8.6)	2 (0.8)	15 (4.8)	2 (1.1)	1 (2.5)
The three years	115 (27.6)	16 (3.1)	63 (17.5)	13 (4.7)	13 (7.3)
Faster-flow spring					
2016	15 (5.9)	4 (1.2)	13 (4.4)	2 (1.1)	1 (2.7)
2017	38 (7.4)	1 (0.7)	25 (5.6)	1 (1.7)	0 (3.2)
2018	20 (7.8)	1 (1.0)	24 (5.9)	2 (1.6)	1 (1.8)
The three years	73 (21.1)	6 (2.9)	62 (15.9)	5 (4.4)	2 (7.7)
Number of cysts per kg of plant					
Total number of cysts	188	22	125	18	15
Dripped plants (kg)	48.7	6.0	33.4	9.1	15.0
Means ± SD	3.8 ± 0.7	3.6 ± 0.7	3.7 ± 0.6	1.9 ± 0.4	1.0 ± 0.4

### Position of metacercariae in the watercress bed

**Figure 2:**
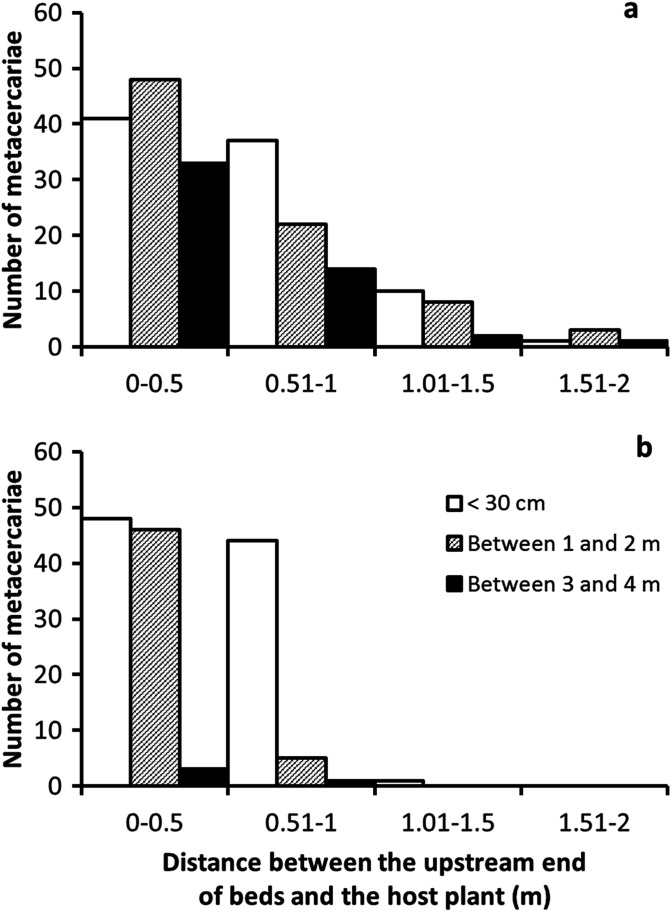
Distribution of *Fasciola hepatica* metacercariae in relation to the distance between the host plant and the upstream end of each bed: (a) sites fed by a slow-flowing spring and (b) those fed by a faster current of water. This distance is expressed as 50-cm sections. The three types of length indicated in the legend (graph 2b) are those of the zone without macrophytes.

Figure 2 shows the distribution of *F. hepatica* metacercariae in relation to the linear distance between the upstream end of watercress beds and the host plants. These values are presented for the two types of beds and the three types of length for the macrophyte-free zone. Most cercariae encysted on the plants in the most upstream part of each bed, usually on those growing on the first 50 cm. No metacercaria was found in the downstream part of each bed. In addition, the distribution of cysts on the plants varied according to the length of the macrophyte-free zone. In beds crossed by a slow current, the metacercariae were found over a distance of 2 m and the number of these cysts decreased progressively from the highest 50 cm upstream to the 1.5–2 m section (Fig. 2a). In beds crossed by a faster current, the metacercariae were located on a shorter length: 1.5 m if the length of the macrophyte-free zone was less than 30 cm, 1 m only when this length was greater. The numerical decrease of metacercariae from the earliest 50 cm upstream was also more marked, especially when the length of the macrophyte-free zone was greater than 1 m (Fig. 2b). The speed of the water flow (*H*_1_ = 22.68, *p* < 0.001) and the length of the macrophyte-free zone (*H*_2_ = 16.98, *p* < 0.001) had significant effects on the distribution of metacercariae in these beds, while the influence of the other factors tested was not significant. Cercariae were able to swim or were carried away by the current up to a mean of 5 m in slow-flow waters before encysting; this distance was only 4 m in faster waters.

Table 3 provides the distribution of *F. hepatica* metacercariae according to the position of their host plants relative to the width of the drainage furrows. These larvae on their host plants were more numerous in the 1–15 cm and 30.1–45 cm sections than in the central one. But the differences between the numbers of metacercariae were not significant.

**Table 3:** Total number of *Fasciola hepatica* metacercariae in relation to the position of the host plant in the watercress bed. The width of the drainage furrow is expressed here in sections of 15 cm each.

Type of watercress bed	Length of the macrophyte-free zone (m)	Number of metacercariae per 15-cm wide section			Total number of metacercariae
		0.1–15	15.1–30	30.1–45	
With a slow-flow spring	0.13–0.24	29	23	37	89
	1.1–1.8	35	27	19	81
	3.1–3.5	23	11	16	50
With a faster-flow spring	0.15–0.29	45	12	36	93
	1.3–1.6	26	7	18	51
	3.0–3.2	3	0	1	4

## Discussion

Among the five species of plants studied, *N. officinale*, followed by *H. nodiflorum* and *Callitriche* sp. in decreasing order of frequency, were the most used by *F. hepatica* cercariae to encyst: a mean number of 3.8 cysts on *N. officinale*, 3.7 on *H. nodiflorum* and 3.6 on *Callitriche* sp. (Table 2). These results are in agreement with the observations reported by Dreyfuss et al. [5] and Rondelaud [20] for the first two species of plants in 59 wild watercress beds on acidic soils for 15 years. In contrast, the mean number of metacercariae is low on *J. effusus* and *V. beccabunga*: 1.9 kg^−1^ and 1.0 kg^−1^ of plant, respectively (Table 2). These two species of plants appear to be little used by *F. hepatica*, with *Callitriche* sp.*, H. nodiflorum*, and *N. officinale* being the most exploited host plants by the cercariae for their encystment. This opinion is based on the observations of Pécheur [16]. Of the nine species that this author proposed to the cercariae of *F. hepatica* under laboratory conditions, only three of them were used by these larvae to encyst.

In both types of watercress beds, the most upstream 50-cm section was the most used by *F. hepatica* cercariae to encyst, whatever the length of the macrophyte-free zone. The number of these larvae then decreased in the remaining 50-cm sections downstream so that the length of the bed with cyst-carrying plants did not exceed 1.5 m or 2 m depending on the type of bed (Fig. 2). These results are more difficult to interpret. Even though the cercariae of *F. hepatica* are more likely to encyst on smooth-surfaced green plants [7, 16], the plants that are found in the most upstream section of 50 cm belonged to one or other of the species studied. As a result, the preference of the cercariae for a specific plant species cannot by itself explain the concentration of these larvae on the plants in this section. Two possibly complementary hypotheses can be proposed to explain this finding. The effort that cercariae provide by swimming over a long distance and in particular in the case of watercress beds located more than 3 m from the nearest snails may reduce glycogen and fatty acids present in the body of these larvae. This could result in gradual exhaustion of these larvae and their encystment on the first plants after entering the bed. However, passive entrainment of these larvae downstream under the effect of the current, forcing them to encyst as soon as they encounter a plant support, cannot be completely ruled out.

According to our results, the metacercariae are far more numerous on the plants present in the peripheral sections of the furrows (0–15 cm and 30.1–45 cm wide) than on those growing in the central section, irrespective of the type of watercress bed (Table 3). The swirls that occur during the water current entry in the watercress bed could be the source of this distribution, by deflecting the cercariae towards the peripheral sections of the furrow, and by causing their fixation and their encystment on plants in these peripheral sections.

In conclusion, plants growing on the most upstream section of a watercress bed in an open drainage furrow were the most used by *F. hepatica* cercariae for their encystment, when the host snail was living around the emergence of a source. The speed of the water current affected the number and distribution of metacercariae in the bed.
